# Nexus Between Demographic Change and Elderly Care Need in the Gulf Cooperation Council (GCC) Countries: Some Policy Implications

**DOI:** 10.1007/s12126-017-9303-9

**Published:** 2017-08-24

**Authors:** Hafiz T. A. Khan, Shereen Hussein, John Deane

**Affiliations:** 1 The Graduate School, University of West London, London, W5 5RF UK; 2 King’s College London, London, WC2R 2LA UK; 3 School of Health Sciences, Faculty of Health, Education and Life Sciences, Birmingham City University, Birmingham, B15 3TN UK

**Keywords:** Human Development Index, Human resources, Gross National Product, Socio-economic, Demographic change, Ageing, Labour market

## Abstract

Population ageing is a phenomenon affecting the whole world. The countries that make up the Gulf Cooperation Council (GCC) are no exception but transitions in population ageing are still in the early stages of the process. With current demographic dividends experienced by the GCC and the rest of the Middle-East, the pace of population ageing will be faster than that experienced by many European countries. The purpose of this paper is to explore the population ageing experience of different GCC countries while situating this within a context of social policies that still at the very early stages of acknowledging such change. We utilise data from sources such as the United Nations and the World Bank, complemented by policy analysis of current age-related social security measures in the GCC. Given the importance of the family aged care system in the region, we consider the implications of changes in family structures, living conditions, and care needs for the elderly. The findings confirm the declining trend in fertility combined with increased life expectancy in all the six GCC countries. However, they highlight that social policy measures focused on the older generations and their care needs are still relatively at the early stages of each country’s policy agenda. The implications of such changes are serious in term of both the demand for and supply of care. Policy-makers need to adapt cohesive social policy strategies that strengthen the complementing relationships between the state, family and wider community as stakeholders in the provision of aged care.

**Electronic supplementary material:**

The online version of this article (doi:10.1007/s12126-017-9303-9) contains supplementary material, which is available to authorized users.

## Introduction

The past decades have witnessed dramatic socio-economic and demographic transitions in many parts of the world. This has caused concern among planners and policy-makers as to what directions they should be going in order to ensure their countries run smoothly while different groups of the population are protected from economic, health and social harm. In this age of globalisation, economic growth has gained priority over so many other important human, family, and social issues. Today we live in rapidly changing societies where science and technological improvements are influencing peoples’ decisions on such issues as education, health needs, marriage decisions and living arrangements among others. Demographic changes, particularly declining fertility rates and population ageing, have profound implications for government policy. These changes prompt the need to speed up sustainable development that is capable of maintaining economic growth while providing certain levels of social security to the most vulnerable in society.

Within the Arab and Middle Eastern context, the family is considered the solid foundation of population growth and has played an important role in human development (Abdelmoneium and Alharahsheh 2016; Hussein and Ismail 2016; Kuhn 2012; Omran and Roudi 1993; UNDP 2016). In current times, with the changing nature of economic development, family norms have sharply changed along with other demographics such as people getting married later, making frequent moves, having fewer children, and living for longer. These changes have impacted on traditional values and cultural practices, in particular on family structures; living arrangements and structural changes in population distribution, all of which are significantly associated with population ageing (Winkler 2002). The Arab family structure is being threatened by all these ongoing changes that have clear implications for living arrangements and elderly care (Abdelmoneium and Alharahsheh 2016; El-Haddad 2003). Researchers are keen to understand the changing nature of family norms in various settings and how this might have influenced other developmental issues (Abdelmoneium and Alharahsheh 2016; Al-Kandari and Crews 2014). Although the dynamics of linkages between population change and socio-economic improvements are evident in some more developed countries (Clausen and Paden 1985); it is, as yet, unclear for some oil wealthy Arab countries particularly in the Middle East. On the other hand, little is known about the impact of socio-demographic changes in Middle Eastern countries on overall quality of life especially of older people living in the region. In fact, there have been some positive developments in various sectors in the Arab world such as in human capital, and to some extent in social protection and health and wellbeing indicators (Al-Kandari and Crews 2014; Hussein and Ismail 2016; Jarallah and Al-Shammari 1999). These factors in turn have been found to influence family structures in Arab societies and consequently on the availability of aged care, particularly in relation to proximity of living arrangements and both internal and external migration. Theoretical notions on causality and how these factors are interlinked have been evident in social science literature (Karagiannaki 2005; Kendall and Anglewicz 2016; Rahman 2010), but have not yet been explored explicitly with regard to Arab societies. The aim of this paper therefore is to assess the overall ageing situation in the Gulf region and to examine the need for elderly care and support.

## Theoretical Overview

Demographic change is a reality in the 21st century and fertility decline is part of the demographic transition that has occurred in most parts of the world (Coleman 2001; Khan 2006, 2014; McDaniel and Zimmer 2013). Socio-economic factors combined with family planning programmes have influenced people to opt for a smaller family size in order to maintain a reasonable quality of life. People move frequently from one place to another in search of jobs or education, for instance, and decide to settle in new places even within the same country. This is an increasingly significant phenomenon influenced by urbanisation and globalisation as well as individual aspiration. Such a trend means that people are moving away from their family members, further challenging the traditional family-based aged care model operating in the majority of the region. Moreover, modernisation has encouraged many people to live alone and away from family responsibilities that may fuel a further erosion of traditional beliefs and values around family structure. As more and more people prefer, or are left with no choice but, to live as part of a nuclear family, the traditional close proximity of living or co-residing within extended family arrangements is declining and expected to decline further. Studies show that co-residence households play important roles in the lives of family members particularly for the older generation (Rahman 1999; Rahman et al. 2004).

The elderly in neighbouring regions of Asia, particularly in developing countries, have traditionally relied heavily on household members for their wellbeing and survival. Households represented the main institution for the distribution of goods and services between generations, and provided the principal vehicle for the expression of age and kinship roles (Bongaarts and Zimmer 2002; Pimentel and Jinyun 2004). Older people from this region also showed a preference to live in their usual homes that included other family members rather than on their own (see for example, Kofor 2006; Leeson 2006; Sokolovsky 2000). Silverstein et al. (2006) found that older parents living in three-generation households or with grandchildren in skipped-generation households in China experienced better psychological wellbeing than those in single-generation households. Other studies conducted in Bangladesh also claim that co-resident households have a positive impact on the wellbeing of older people (Rahman 1999; Rahman et al. 2004). The gradually declining pattern in co-residence along with declining geographical proximity between older parents and adult children have brought new concerns about social support in many Asian countries including the Philippines, Thailand, Indonesia and Bangladesh (Knodel and Ofstedal 2002; Rahman 1999; Schroder-Butterfill 2003). The extended family household has, for centuries, provided the basis for the traditional family support system that encompassed caring roles across generations. The care roles included grandparents providing child-care while parents (middle generation) provided various forms of elder care including financial, emotional and personal care and support. Research carried out in Asia reveals that co-residence with an adult child is indicative of upward flows of social support (Knodel and Debavalya 1997). Moreover, co-residence with children in some countries remains virtually the only source of support for older people needing care. The situation is very similar in the majority of Muslim countries in the Arab Region, particularly the challenges that family caregivers face in providing care for older people (Abdelmoneium and Alharahsheh 2016; Hussein 2009).

Studies conducted over the years recognised that changes in family structures had implications for the wellbeing of elderly people (Du et al. 2004; Jiang 1995; Wang and Li 2007; Zimmer 2003). The younger generation has traditionally taken care of older people as part of intergenerational solidarity but this pattern is changing quite dramatically (Hussein and Ismail 2016; Khan 2014). The non-economic informal support system for the elderly is being challenged where low fertility rates mean fewer children leading to a narrowing of the pool of children sharing responsibility for parental support. Conversely, with increasing longevity, the proportion of the elderly living in households demanding physical and financial support from their children is on the increase. On top of this, the migration of adult children increases the geographic distance between parents and their offspring, making it more difficult for offspring to provide the necessary support to their parents in person (Bernstein 2002; Du et al. 2004). A gap between the demand for and supply of care support for the elderly thus emerges at the household level, which leads to a reduction in the quality and quantity of the availability of informal elderly personal care. Low fertility, on the other hand, may be linked to a reduction of household investment in children so enhancing the ability of adult children to support their parents through formal, paid, care services. Out-migration brings new occupational opportunities for adult family members, helps to increase their wage income and improves their ability to provide monetary support for their parents (Du et al. 2007). Additionally, women who have traditionally provided care for older parents and parents-in-law are increasingly staying for longer in education and then participating in the labour market. While the feasibility of financial support to older parents may increase within such dynamics, the availability of hands-on-care and emotional support is challenged.

The scale of change in family structures and living arrangements leaves much uncertainty about the role of the family for elderly wellbeing. Past studies have suggested that changes in family size and living arrangements decrease the quality of care and support among the elderly and exacerbate the risk of poverty (Bernstein 2002). The declining trend of support ratio for older people globally will make it harder to secure future demands for support and care and may have an adverse effect on the overall quality of life (Hussein and Khan 2012, Khan 2014).

**Fig. 1:**
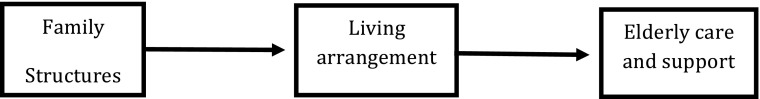
A conceptual framework for elderly care model

There are indications of a causal link between family structures and living arrangements that has a direct influence on elderly care within the household. Today’s multi-generational society where four generations of a family can be alive at the same time, allows a real opportunity for families to share positive experiences and values. Existing literature has contributed to the understanding of such dynamics, but it has shortcomings. The methodology, for instance, tends to be descriptive; few studies have modelled the potential linkage between family changes and the care and support needs of the elderly; existing data is inadequate to address the issues of whether family changes may be positively and/or inversely associated with elderly wellbeing. Thus, the main purpose of this paper is to investigate linkages between demographic change and the need for elderly care in the Gulf Corporation Council (GCC) countries. In order to capture a broader picture, we used a number of demographic factors including living arrangements in the household and the extent to which they varied across the GCC geographical settings. A conceptual model is put forward to indicate how it works in today’s society (Fig. 1).

Many issues remain unaddressed in research up to now. For example,

- What is the current status of support for the elderly in relation to family changes?
- To what extent do family changes relate to the dimensions of living arrangements?
- What are the determinants of elderly wellbeing, and in particular, what roles do changes in family structures and living arrangements play in elderly care and support?

This paper attempts to answer these questions, as much as available data allows, within the conceptual framework and examines them empirically for each GCC country. The comparisons will help to form an understanding of the situation across the six countries involved in the study. The remainder of this paper looks into the setting, sources of data and methods employed for analysis. A detailed discussion on the results is provided in a separate section and this is followed by some conclusions and policy implications.

## Methodology

### The Setting

The Gulf Cooperation Council (GCC) was established in May 1981 by six Middle Eastern countries: Kingdom of Bahrain, State of Kuwait, Sultanate of Oman, State of Qatar, Kingdom of Saudi Arabia and United Arab Emirates (UAE). The GCC was initially formed to achieve unity among member states based on common objectives with regard to economic development, politics and religious interests. All six-member states are geographically adjacent with the territory of the Kingdom of Saudi Arabia and they share many common values including political and cultural identities.

**Fig. 2:**
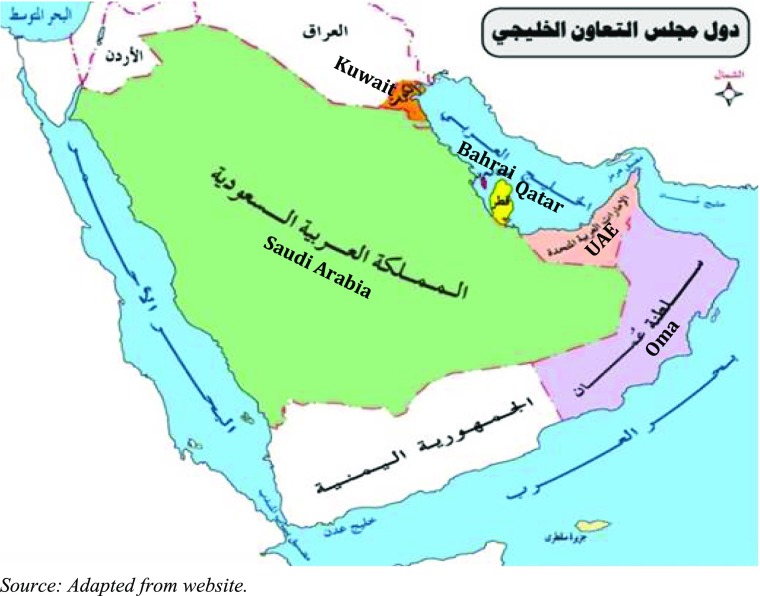
Geographical map of GCC countries

Population growth and human resource development are key common agenda items in the plans of almost all governments across the world and GCC countries are no exception. For the last two decades, the GCC countries have developed their economies faster than any others in the Middle East or any neighbouring Asian countries. Figure 2 shows the close geographical locations of the GCC countries and how important the union is in terms of mutual cooperation, economic activities, political and regional stability, and above all, for sustainable development.

Population growth in many Arab countries creates considerable demands on the economic, social and educational infrastructure (Anderson 2007; Hussein and Khan 2012; OxGaps 2013). Good policy requires adequate sources of information, rigorous data collection and quality official statistics. Demographic information is key in enabling better planning and policy-making decisions.

### The Data

A review of suitable comparative indicator across the six GCC countries revealed the most suitable to be from secondary sources such as GCC official statistics, the World Bank, and UN population perspectives to make a comparative study across all six GCC countries. While these data and indicators might provide somewhat limited information related to the specific indicators collected, they offer a number of advantages, including accuracy, accessibility and comparability across the six nations. The 2012 revisions of the world population perspectives are used mainly to compare the dynamics of socio-demographic changes in the region using various indicators at the aggregate level of individual countries. Some important demographic variables such as age distribution of populations, total fertility rates, life expectancies at birth and socio-economic variables including education, labour participation and human development indices were examined in order to develop the scenario analysis for the selected countries. We explored available GCC data for living conditions, family structures and elderly care and support but unfortunately such data were not available for a cohesive study. Therefore, the present study can only raise the importance of such a study and future research can investigate our proposed research hypotheses. Despite the limitations we aimed to establish relationships between demographic changes and the consequences for elderly care in the region. To this end, available data is analysed and tables and graphs are provided for comparison.

## Results and Discussion

### Socio-Demographic Changes in the GCC Region

**Fig. 3:**
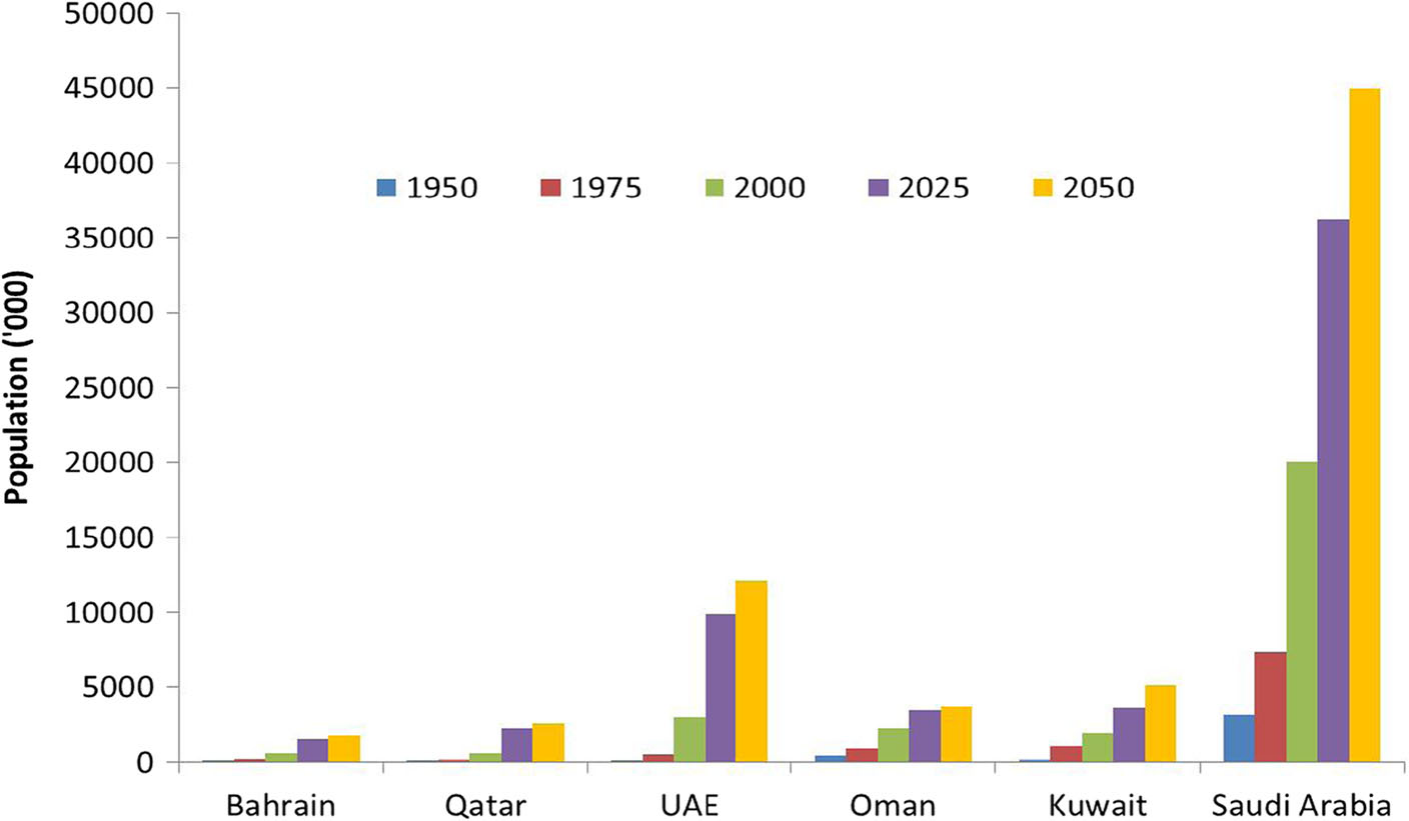
Population trends in GCC countries (1950–2050)

Population growth has been observed in most of the GCC countries since the 1950s and this trend is projected to continue to 2050 (Fig. 3). The analysis shows that Saudi Arabia has the highest population size followed by UAE. According to statistics produced by the UN, in 2015 the total recorded population numbers (in millions) were: 1.4 in Bahrain; 2.0 in Qatar; 8.3in UAE; 3.0in Oman; 3.0 in Kuwait and 30.5 in Saudi Arabia. The total population in the GCC region in 2015 was estimated to be around 45.6 million. This indicates that the size of the population in Saudi Arabia is more than double (62%) the size of the populations of the rest of the GCC countries added together. By 2050, the populations are likely to increase at different rates in the region. The analysis shows that populations will increase 2.82, 4.42, 4.01, 1.64, 2.66 and 2.24 fold in Bahrain, Qatar, UAE, Oman, Kuwait and Saudi Arabia respectively. Qatar is projected to have the biggest increase. There are also age structural transitions happening in the distribution of populations and in age and sex.

**Fig. 4:**
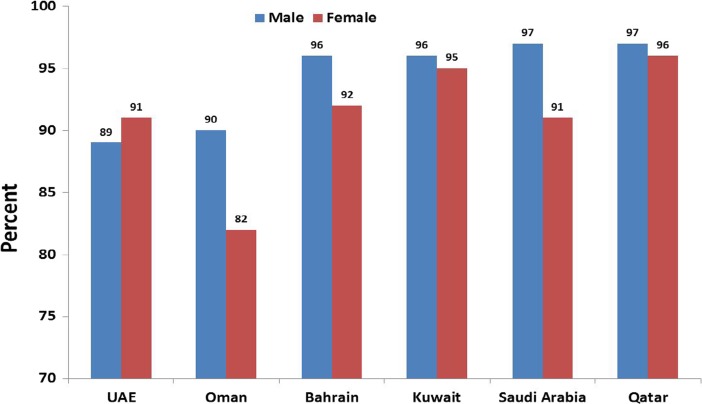
Literacy rate - population 15 years and over who can read and write (2005–2013)

Demographers are increasingly studying population related issues that predominantly focus on human development *vis-à-vis* socio-economic, demographic, political and cultural contexts in the region (for example, Ananta et al. 2005; Bongaarts and Zimmer 2002; Coleman 2001; Grundy 1997; Hermalin 1997; Kent 2005; Knodel et al. 2000; Raeside and Khan 2007). The literacy rate is one indicator that could be used to understand the overall position of a country. Adult literacy is lower for females than males (Fig. 4) with a very significant gender difference in Oman. Qatar has made significant progress in improving literacy rates for both genders. It is well documented that female education has a significant impact on age at first marriage as well as on decision-making roles in families and on fertility (Khan and Raeside 1994, 1997; Caldwell et al. 1992).

**Fig. 5:**
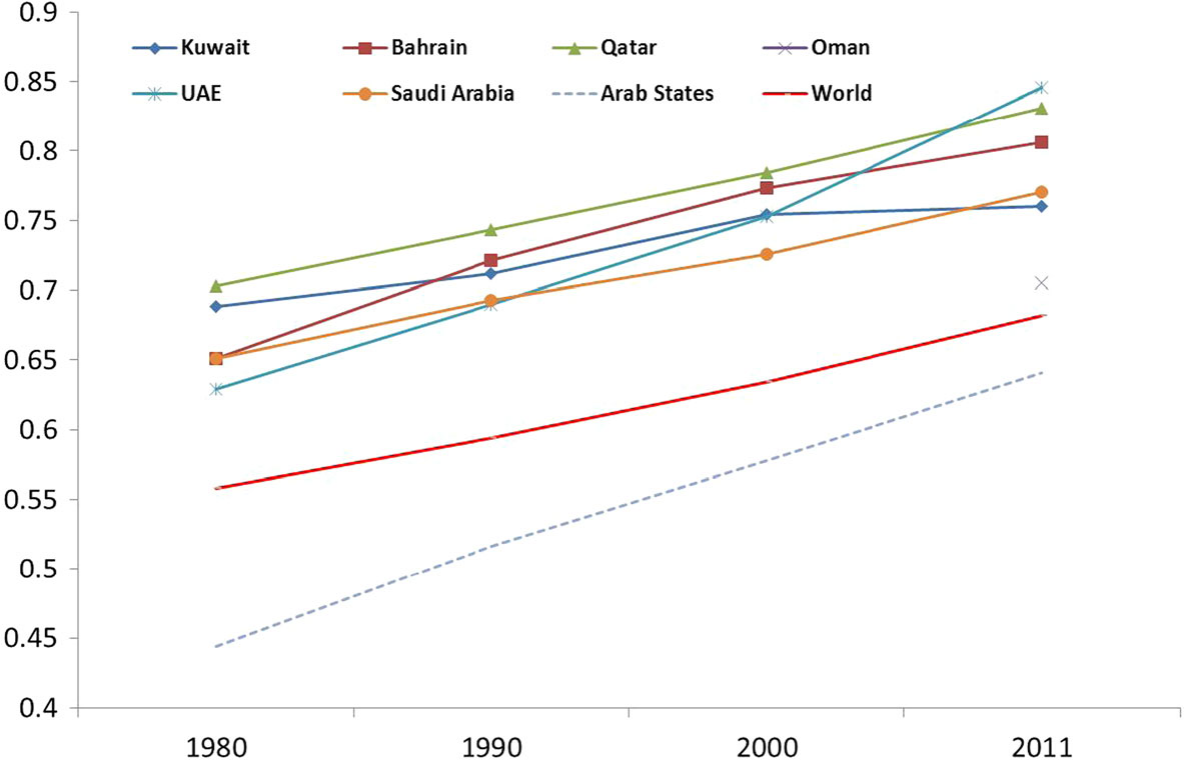
Human Development Index for GCC countries

The Human Development Index (HDI) is widely used to compare the socio-demographic situation in many countries. This index is a composite of literacy rates, Gross National Product (GNP) per capita and life expectancy at birth among other measures (UNDP 2008). The higher the literacy rate then the better the HDI. According to the HDI, all GCC countries have been improving their rank positions since 1980 with Qatar ranked at the top of the list as shown in Fig. 5. All GCC countries ranked much higher than the world average. The HDI indicator gives a clear message about the overall improvement of human capital. This indicator is also linked with demographic variables such as fertility and mortality. For example, Bongaarts and Watkins (1996) found that a strong negative correlation exists between HDI and the total fertility rate (TFR) in a country. It is expected that people’s aspirations increase with the gradual improvement of HDI and include a better quality of life, freedom of speech, social justice and above all human rights.

**Fig. 6:**
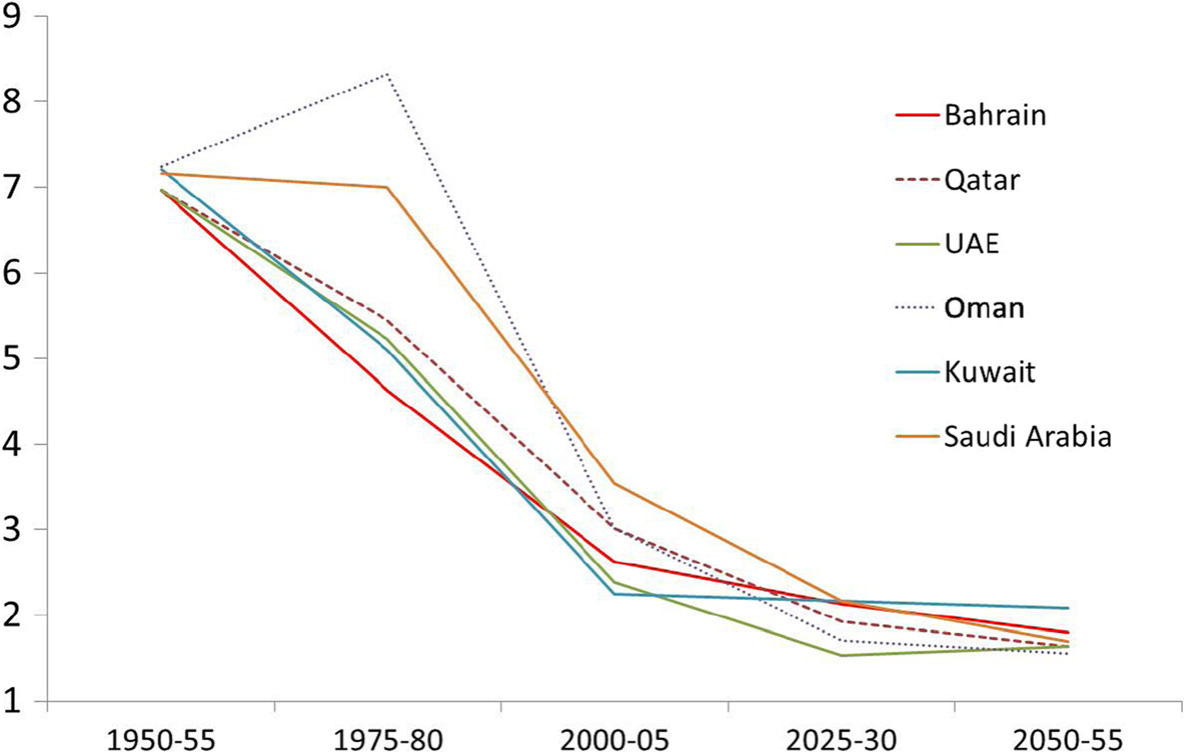
Trends in actual and projected total fertility rate in GCC countries (1950–2050)

A dramatic change has taken place in the fertility levels in all the GCC countries (Fig. 6). Fifty years ago, the total fertility rate (the average number of children a woman would have over her reproductive life) was almost at seven in all the GCC countries but by the mid-1970s, a big variation had started to emerge. During the 1980s and 1990s, fertility rates began to fall quickly in all the GCC countries apart from Oman and Saudi Arabia. The variation in fertility rates has gradually reduced in all countries since then and today it has come down to as low as 2 to 3 children. Saudi Arabia seems to be exceptional in having a higher fertility rate compared to the rest of all the other GCC countries. More research is needed to explore the reasons for this. It is predicted, however, that by 2050 all GCC countries will achieve the threshold of below replacement levels of fertility (which is TFR = 2.1). This process will have major ramifications on the age structure of the population in the GCC region.

Such reduction in TFRs has been combined with changes in nuptiality patterns across the region. Within the Arab region, unlike the historical experience of Western Europe, fertility continues to be governed by marriage with extra-marital childbearing not having been observed in the region so far (Engelen and Puschmann 2011). Nuptiality changes include a decline in the prevalence of early marriage of women, which has traditionally been the norm in the region. For example, the percentage of married women in the age group 15–19 has fallen from 57% in 1975 to only 8% in 1995 in the UAE (Rashad et al. 2005). The universality of marriage and mean age of marriage have also been changing in the region with the singulate mean age at marriage for women in Oman, for example, rising from 19.2 years in 1988 to 23.5 years in 2000 (Oman Ministry of Health 1989, 2000). However, many marriages remain governed by traditional family norms of kinship and the strong influence of parents in spousal selection particularly in the Gulf region where over half of the total marriages in Oman remain consanguineous (Islam et al. 2013).

**Fig. 7:**
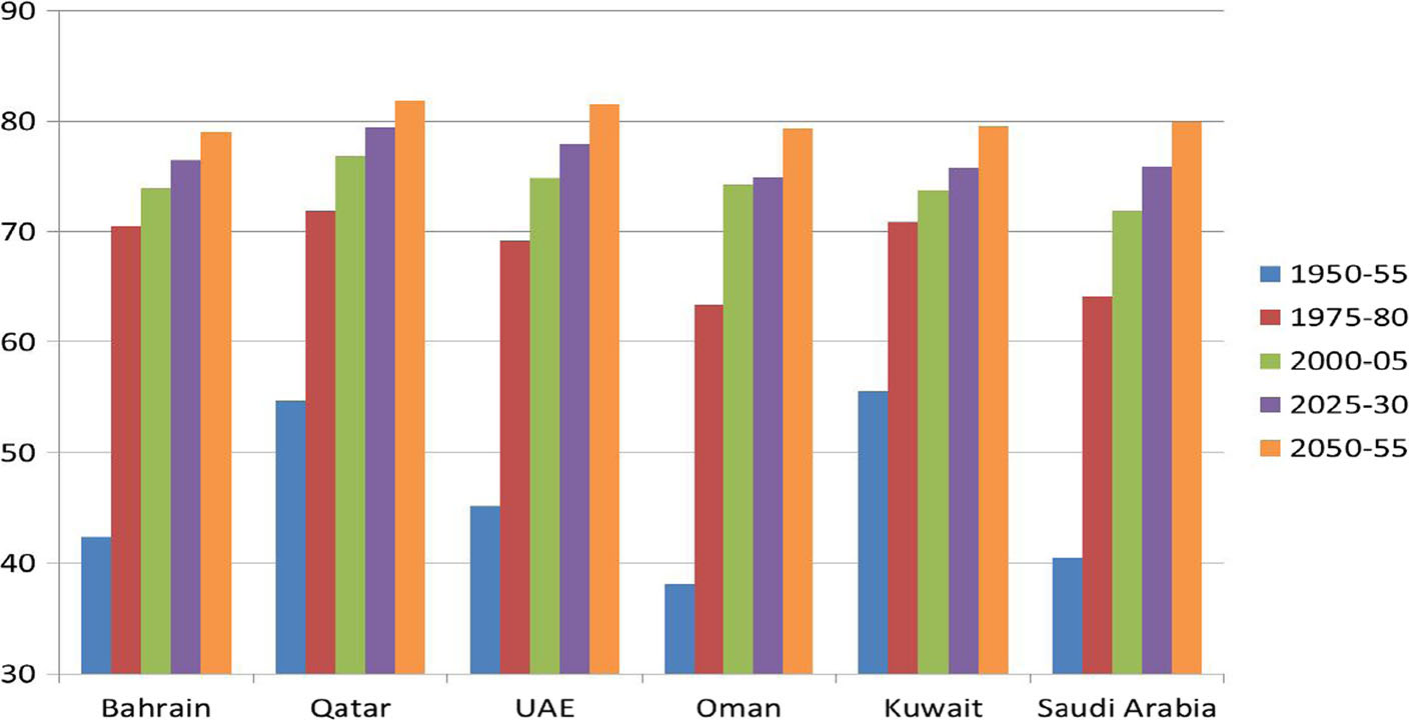
Actual and projected average life expectancy at birth in GCC countries, 1950–2050

All GCC countries have made huge improvements in reducing mortality as well as morbidity levels, which in turn has had a tremendous impact on life expectancy. In 1950, life expectancy at birth was estimated to be almost 40 years on average in GCC countries but by 1970 it had risen to over 70 years. In 2025, the UN has projected a life expectancy at birth in almost all the GCC countries of 75 years increasing to almost 80 years by 2050 (Fig. 7). Longevity is a good indicator that GCC countries are heading towards an ageing society. Medical and technological improvements have helped to make living longer a factor of the modern era but longevity might also create new issues and concerns for societies if preparations are not made for tackling the new challenges associated with ageing and ill-health at very old age.

### Demographic Ageing in GCC Countries

Ageing is a bi-product of three main drivers of demographic change: fertility, mortality and migration. Since all these drivers were found to have changed in the GCC countries then it can be expected that the age structural composition of their populations will start to change as well (Khan 2006). This means that from having a wide youthful base, the population pyramid will move to a more rectangular mature shape by the year 2050. Given all the present circumstances, the population of the GCC region is ageing because of a demographic transition from high fertility and mortality to relatively low fertility and moderate mortality rates. The aged population will continue to grow and this presents a number of issues related to their status and roles; care and living arrangements; health and social support and overall wellbeing (see for example, Khan and Raeside 2005, Khan et al. 2013, Khan 2014; McDaniel and Zimmer 2013). Here elderly is defined as the proportion of the population aged 60 years and over and it is commonly used as an ageing index for developing countries.

Table 1 shows the trend of two population groups aged 60+ years and under 15 years. It shows that the younger population is declining over the period 1950–2050 while the elderly population is significantly increasing over the same period. The ageing index is a ratio between the population aged over 60 years and below 15 years and this index has increased in an unprecedented manner in the region. It is predicted that by 2050, the highest ageing index will be observed at 143.7 for the UAE. For Oman, the ageing index is found to be unexpectedly lower at 39.1. There are two possible explanations for this: the quality of data may not be good enough and changes could be happening more quickly within a very short time.

**Table 1:** Ageing index for GCC countries (1950–2050)

Country	Year	% population 60+ years	% population < 15 years	Ageing index
Bahrain	1950 2000 2050	4.6 3.8 28.1	43.3 30.8 13.4	10.6 12.3 137.2
Qatar	1950 2000 2050	5.7 3.0 42.7	42.3 25.9 9.6	13.5 11.6 104.7
UAE	1950 2000 2050	5.7 1.7 34.0	42.3 25.4 11.2	13.5 6.7 143.7
Oman	1950 2000 2050	5.0 3.7 35.7	42.3 37.2 14.7	11.8 9.6 39.1
Kuwait	1950 2000 2050	4.5 4.7 16.3	36.1 45.7 19.4	12.5 10.3 130.4
Saudi Arabia	1950 2000 2050	5.6 4.7 25.3	42.0 38.4 15.9	13.3 12.2 55.8

Source: World Population Ageing 1950–2050. Population Division, DESA, United Nations

Ageing is a cause for celebration and is an achievement for future generations but it is also associated with several socio-demographic changes:

i. Increased trend for labour participation of ‘traditional’ informal care givers (usually women)
ii. Increased trend in ‘lone-residency’ at old age (usually women)
iii. Higher widowhood prevalence among older women; offspring migration (internal or international); co-residency and social changes etc.
iv. Increased chance of living with dementia particularly among women
v. Changes in ‘expectations’ of old age and quality of life
vi. Long term-care needs
vii. Increased importance of ‘formal’ care.

Old age dependency ratio is defined as the proportion of people aged 60+ to those aged 15–59 years of age. It indicates how many older people needed to be supported financially by the working age group in an economy. Table 2 shows that the old age dependency ratio will increase in all GCC countries by 2050 leading to an increased burden for young wage earners and the government.

**Table 2:** Old-age dependency ratio (Proportion of population 60+ compared to 15–59 years

Country	Year	% population 60+ years	% population 15–49 years	Old-age dependency ratio
Bahrain	1950 2000 2050	4.6 3.8 28.1	53.1 65.3 58.4	8.7 5.8 48.1
Qatar	1950 2000 2050	5.7 3.0 42.7	52.1 71.1 47.7	10.9 4.2 89.5
UAE	1950 2000 2050	5.7 1.7 34	52.1 73.0 54.7	10.9 2.3 62.2
Oman	1950 2000 2050	5.0 3.7 35.7	52.7 59.1 49.6	9.5 6.3 72.0
Kuwait	1950 2000 2050	4.5 4.7 16.3	59.4 69.6 64.4	7.6 6.8 25.3
Saudi Arabia	1950 2000 2050	5.6 4.7 25.3	52.4 56.9 58.8	10.7 8.3 43.0

**Fig. 8:**
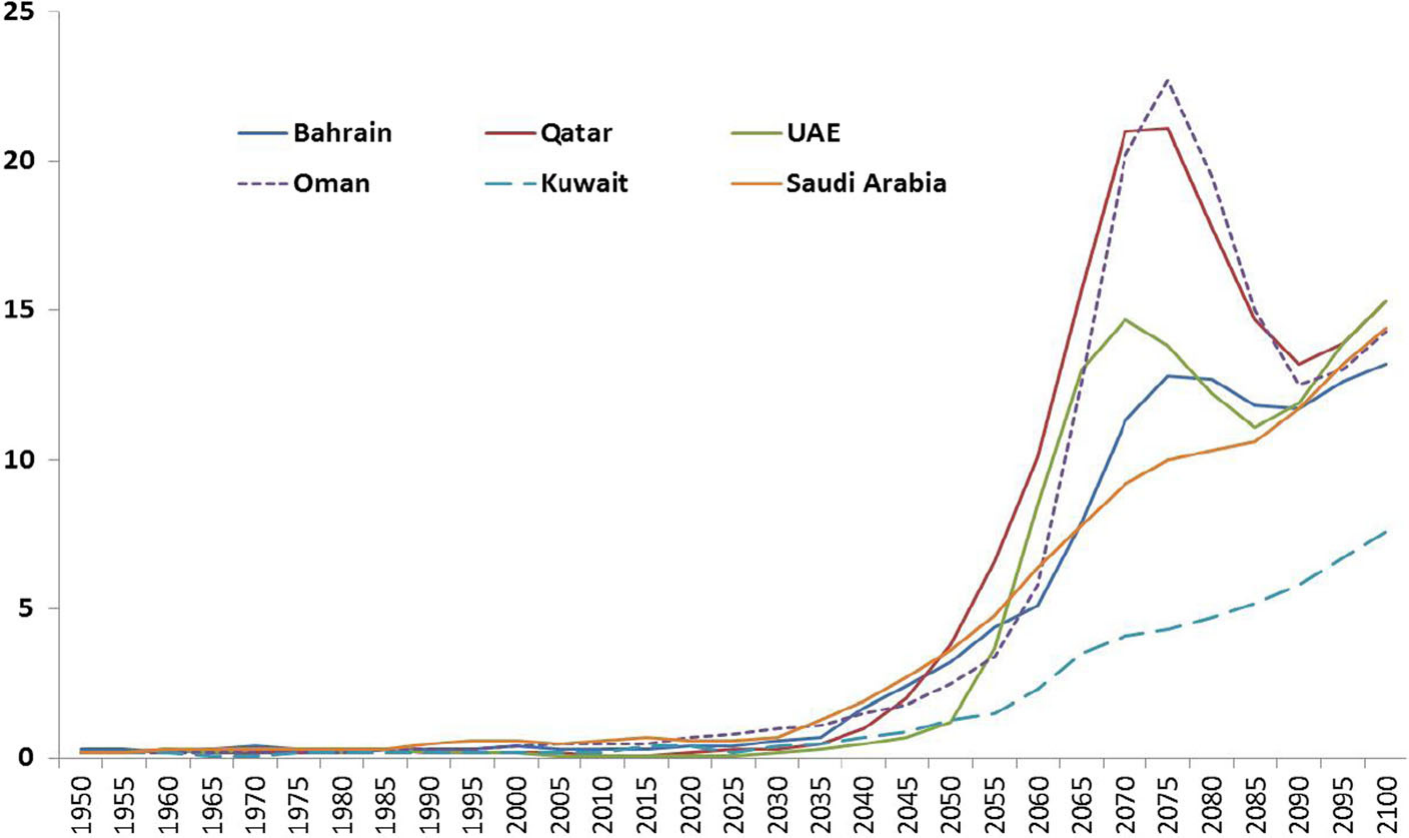
Percentage of 80+ populations in GCC countries (1950–2100)

Given that many people would still be active at age 60 years, we examine the proportion of the population aged 80+ years in all the GCC countries. The analysis shows that this proportion started to increase from 1990 and that this trend is predicted to continue (Fig. 8). It is highly likely that up to 3% of the total population will be 80+ and a large majority of them will be women because life expectancy for women is higher than for men. High levels of morbidity including different long-term conditions that limit daily activities are observed among people aged 80+ (Al-Kandari and Crews 2014; Khan 2017). As expected the risk of disability particularly dementia is likely to increase among this age group. Long-term care needs thus would require support from informal caregivers that is, the families, as well as from the wider health system including emerging formal long-term care services. Thus, long-term planning and preparation is essential to cope with the demands of an ageing population. The question is whether the GCC countries are well prepared for this or not. Similar to other countries, the future demand for health and social care particularly among the elderly will require a considerable supply of trained care services to meet such an escalating demand (Hussein 2009; Hussein and Khan 2012).

### Old-Age Policies in the GCC Countries

The facts and figures of the GCC countries demonstrate that life expectancy is increasing although the state statutory pensionable age is still very low compared to countries across the world. The GCC economy is largely energy dependent and governments do not have any policies towards their older populations. As can be seen from Table 3, the per capita GDP in all the countries is reasonably high and questions remain as to how services in older age can be provided without sustainable economies within these countries. Thus, systematic research is urgently needed on the care workforce supply side in order to aid these countries in coping with the care needs of their ageing populations.

**Table 3:** Demographic and other statistics related to social security for GCC countries, 2012

Country	Total populations (millions)	Life expectancy at birth		Statutory pensionable age		GDP per capita (US$)
		Men	Women	Men	Women	
Bahrain	1.3	74.7	76.1	60	55	27,700
Kuwait	2.7	74.0	75.9	50	50	41,700
Oman	2.8	71.4	76.4	60	55	27,600
Qatar	1.8	78.7	78.2	60	55	91,379
Saudi Arabia	27.4	73.2	75.6	60	55	23,480
U.A.E	7.9	76.0	78.0	60	55	43,048

SOURCES: United Nations Population Division, Department of Economic and Social Affairs. World Population Prospects: The 2010 Revision Population Database, available at http://esa.un.org/unpd/wpp/unpp/panel_indicators.htm (2010); Human Development Report 2011, prepared for the United Nations Development Programme (Gordonsville VA: Palgrave Macmillan, 2011); U.S. Central Intelligence Agency. The World Factbook, 2012 (Washington D.C.: Central Intelligence Agency, 2012), available at https://www.cia.gov/library/publications/the-world-factbook/index.html

In realising the urgency and need for support of older people, all GCC countries have taken some action and there are provisions in place. This includes state social security (see Table 4) for example, so that people can get benefits. However, there is uncertainty around the future support needs of an ageing population when a country’s economy faces a difficult time. Policy-makers should be thinking about this now before it is too late.

**Table 4:** Description of old-age and disability social insurance systems in the GCC countries

Country	First and current law	Coverage	Basic qualifying conditions	Basic benefits
Bahrain	1976	Bahraini employed persons in establishments with one or more employees or working in any GCC. Exclusions: Household workers, certain groups of agricultural employees, casual workers, temporary non-citizen workers, and other groups as specified by law. Special system for public-sector employees.	Old-age pension: Age 60 (men) or age 55 (women) with at least 10 years of coverage. Early pension: Regardless of age with at least 20 years of coverage (men) or 15 years of coverage (women). Lump-sum compensation for prolonged service: Paid if the deceased had more than 40 years of contributions.	Old-age pension: The monthly pension is 2% of the insured’s monthly average earnings in the last 2 years multiplied by the number of years of contributions. The minimum pension is the insured’s average contributory wage during the last 2 years or 180 dinars a month, whichever is less. The maximum pension is 80% of the insured’s average earnings plus an additional 10% of the pension.
			Old-age settlement: Paid at age 60 (men) or age 55 (women) if the insured person does not meet the contribution conditions for the normal old-age pension.	Old-age settlement: A lump sum is paid of 15% of the insured’s average monthly earnings in the last 2 years multiplied by 12 times the number of years of contributions plus 5% interest from the date coverage stops until the date the settlement is paid.
			Disability pension: The insured must be younger than age 60 (men) or age 55 (women) when the disability began. The insured had at least 6 consecutive months of contributions immediately before the disability began or 12 non-consecutive months of contributions with 3 months immediately before the disability began.	The pension is 44% of the insured’s average monthly earnings in the last year of contributions before the disability began or 2% of the insured average earnings during the last year of contributions multiplied by the number of years of contributions, whichever is higher.
Kuwait	1976 (civilians); 1980 (military) and 1992 (supplementary	Basic system: Public, private and oil sector employees, self-employed and military personnel.	Basic system: Age 50 with at least 15 years contributions (age to increase gradually to 55 by 2020). Age 48 with at least 20 years of contributions for men and women with no children. Age 43 with at least 15 years contributions for married women and women with children. At any age with at least 20 years contribution. At any age with at least 15 years of contributions for women with caring responsibilities (husband or children, but not parents, with disabilities).	Basic system: 65% (75% for military personnel) of insured’s last monthly earnings (min 230 and max 1250 dinars) or the average monthly insured income in the last 3 years for self-employed, plus 2% for each year of contribution exceeding 15 years up to 95% of earnings (100% for military personnel)
		Supplementary system: Employees with monthly earnings over 1250 dinars	Retirement is necessary, except if moving from the public to the private sector, with certain requirements.	Supplementary system: Accrued sum in the insured’s account divided by a fixed amount varying from 202 to 120 dinars according to age.
			Supplementary system: Paid at the same time as the basic old-age pension	
Oman	1991 (social insurance), implemented in 1992	Citizens of Oman aged 15 to 59 employed in the private sector under a permanent work contract or working in one of the GCC countries	Old-age pension: Age 60 with at least 180 months of paid contributions (men) or age 55 with at least 120 months of paid contributions (women).	The pension is 2.5% of the insured’s average wage in the last 5 years of employment multiplied by the number of full years of contributions.
			Early pension: Age 45 to 59 with at least 240 months (men) or 180 months (women) of paid contributions.	The minimum pension is 100 riyals.
				The maximum pension is 80% of the pensionable salary.
				Early pension: The pension is reduced according to age and gender. For men, the reduction is from 6% (age 59) to 30% (age 45); for women, the reduction is from 7% (age 54) to 25% (age 45).
Qatar	2002 (retirement and pensions), implemented in 2003	Public-sector Qatari employees, some categories of private sector workers, and Qatari working in one of the GCC countries.	Age 60 (men) or age 55 (women) with 15 years contributions.	5% of insured’s average gross earnings in the last 5 years before retirement is paid for each year of contributions
		Exclusions: self-employed persons; and household, family and foreign workers	Early pension: Age 40 with 15 years contributions	The minimum monthly pension is 75% of the insured’s gross monthly earnings and the maximum is 100%
		Special system for military personnel		Early pension is reduced by 2% to 2.5% for each year taken before the normal retirement age
Saudi Arabia	1969 (social insurance), implemented in 1973.	Private-sector and some categories of public-sector Saudi workers.	Age 60 (men) or age 55 (women) with at least 120 months of paid or credited contributions (credited contributions must not exceed 60 months).	The pension is 2.5% of the insured’s average monthly earnings during the last 2 years for each year of contributions, up to 100%.
	2000 (social insurance), implemented in 2001	Voluntary coverage for persons who are self-employed, are working abroad, or no longer satisfy the conditions for compulsory coverage.	Age 55 (men) with at least 120 months of contributions if in arduous or unhealthy work.	The average monthly earnings used to calculate benefits must not exceed 150% of the insured’s monthly earnings at the beginning of the last 5-year contribution period.
		Exclusions: Agricultural workers, fishermen, household workers, family labour, and foreign workers.	At any age with at least 300 months of contributions and if no longer covered by the program.	If the insured’s monthly earnings decrease during the last 2 years before retirement, special provisions apply to adjust the average monthly earnings used to calculate benefits.
		Special system for civil servants and military personnel. Under certain conditions, former contributors under the civil and military scheme may request to have contribution periods credited toward the public social insurance scheme.	Retirement from covered employment is necessary.	The minimum pension is 1725 riyals a month.
U.A.E.	1999 (pension and social insurance)	UAE nationals in private or public employment	60 M/F for Emiratis and 65 for Non-Emiratis	Retirement pension: percentage of final pensionable salary based on the number of completed years of service

Table 4 provides an overview of old age support policies and benefits in the GCC countries. It shows that these countries can be characterised as having primarily residual social welfare systems that provide old age support subject to formal and continued employment. This then excludes many, particularly women, who may rely on informal labour market participation (Al-Kandari and Crews 2014; Khan 2014). These systems are also preferential to public sector and military personnel. A number of laws governing social support at old age are as recent as 2002 in some of the countries such as Qatar. Such residual social welfare systems implicate a heavy reliance on family or community-based social support in relation to aged care. This is especially true for those members of the population who do not have access to welfare benefits that are based on a record of formal employment-based social insurance contributions. This reliance on kin and community-based support is especially prominent in aged care where the state exercises little legislative power and there are no formal systems to support families or individuals who support their elderly parents.

## Conclusion and Policy Implications

This study reveals that dramatic population changes in the GCC region have been caused by a combination of demographic, economic and societal factors. This process has been further propelled by the continuous improvement in human capital, technology, social development, and the use of modern healthcare techniques. Hussein and Ismail (2016) report that there is an on-going trajectory in population structures taking place in the entire Arab World that needs to be addressed with appropriate policy actions.

The success of a country, in meeting the needs of its elderly population, largely depends on having clear and targeted visions and long-term policy initiatives that in turn require rigorous action plans based on population information alongside a regular cycle of high quality data collection. It could be argued that the recent Arab Spring was caused by the youth ‘bulges’ in the populations living in the Region. The new younger generation are different from those of the past because, as citizens of the 21st century, their expectations, aspirations and thinking could be heavily influenced by globalisation. The Arab Spring needs a thorough investigation from a demographic point of view. It has been demonstrated how demographic challenges are often embedded within the socio-economic, cultural and political systems of a country. What is less appreciated is the pace of demographic ageing that the majority of the Arab world is likely to face within the coming decades. This is a result of the ageing of the same youth ‘bulges’ who are likely to enjoy high life expectancy and enter old age in large numbers.

More attention needs to be given to the socio-demographic changes in Middle Eastern countries and the subsequent human resources that will be required to deal with them. The mechanisms of population change are evident as fertility is falling dramatically in all the GCC countries whilst at the same time there is a dramatic increase in life expectancy and significant changes in family formation structures. These are the main determinants of future population changes and ageing for the GCC countries as well as the sustainability of a family-based aged care model. It is possible for these countries to use their large ‘bulge’ of youth by investing in people in order to build capacity in a strategic and targeted manner. The region is currently not well prepared to deal with an ageing population in the longer term. The GCC countries need to address this issue seriously by engaging with stakeholders including academia and Non-Governmental Organisations (NGOs) in order to devise and implement policy programmes. There are several challenges ahead including ensuring that there are reliable data for population projections; funding and resources for the provision of elderly care services; dealing with significant health burdens (e.g., disability); providing health treatments and raising awareness of ageing societies.

This study indicates a clear scenario in relation to changes in family structures across all GCC countries and how these continued changes could affect living arrangements and the future care support needs of elderly people. This study also provides new insights into family and old age support that are emerging issues for the continued security and wellbeing of the family unit. Also highlighted is the increasing demand for elderly care in the GCC countries as is the question of who is going to provide that care and support. Khan et al. (Khan et al. 2013) claim that it is the individual who has to bear his or her own expenses for any sort of care support in later life in developing countries and they found strong evidence to support this claim. If it applies in other countries then the GCC should think seriously about the issues without any further delay. For instance, GCC countries could consider targeting their large youth ‘bulges’ by providing them with proper training and raising their awareness about the changes; they could also start to encourage older people to become involved in various charitable and social activities that may help nation building as well as enhance their own physical and mental wellbeing.

With limited data sources, Hussein and Ismail (2016) have indicated how the Arab region can prepare for the demands that come with an ageing population, particularly with regard to the health care required to ensure a sustainable society. More research is needed in order to map out the key issues related to ageing in the GCC countries that can then be used for preparing policy plans on how these countries can start to tackle those issues.

Although we do not discuss in this paper the role of technology and its potential impact on globalisation, migration or indeed long term care provision, their role in future dynamics should not be ignored and further research is needed to assess their suitability within the Arab region. Similarly, the potential impact of the use of social media on the dynamics of elderly care, with more and more people in developing countries gaining access to the internet and mobile phones for instance. At this stage, there is no available information on these issues in the region, however, possible strategies, both for now and in the future, to address elderly care in the region will probably involve technology and electronic communications. They can be used for helping to combat isolation, improve access to health/social care and to complement hands-on care as well as help relieve pressure/stress on family caregivers whether they live with the older generation or away from them.

Based in the analysis, the following recommendations are suggested for policy-makers in the region:

- Ageing issues should be prioritised and taken seriously in the GCC countries;
- The need for building awareness among populations about people, place and society;
- The gathering of reliable data to help facilitate effective planning and to meet projected demand;
- Developing policy and strategies to address issues of care needs, awareness raising and capacity building;
- Building capacity of the formal and informal care sectors;
- Realising the necessity of developing a health and social care workforce that is appropriate to meet the needs of an ageing society;
- Enhancing the role that community-based care and older people’s activities can play in society.
- Incorporating IT based strategies in addressing future health and social care in the GCC region.

A multi-country, multi-agency approach to these major issues is clearly required and universities, research centres and civil society groups could have a key role in planning and evaluating any pilot projects.

## Electronic supplementary material

ESM 1 (PNG 69 kb)
