# Supplementary figures and images for: Nexus Between Demographic Change and Elderly Care Need in the Gulf Cooperation Council (GCC) Countries: Some Policy Implications
Source: Ageing Int. 2017 Aug 24;42(4):466–87. doi: 10.1007/s12126-017-9303-9 (PMC5702386; doi:10.1007/s12126-017-9303-9)

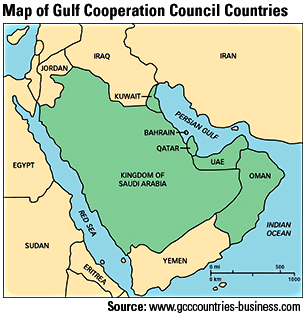

Supplement: Supplementary file 1 — (PNG 69 kb) [file 12126_2017_9303_MOESM1_ESM.png]
